# Association between Sleep Duration and Symptoms of Depression Aged between 18 and 49: The Korea National Health and Nutrition Examination Survey (KNHANES Ⅶ) from 2016 to 2018

**DOI:** 10.3390/healthcare10112324

**Published:** 2022-11-20

**Authors:** Sung-Yong Choi, Ji-Eun Han, Jiae Choi, Minjung Park, Soo-Hyun Sung, Angela Dong-Min Sung

**Affiliations:** 1 Seoul Metropolitan Government Big Data Division, Seoul 04524, Republic of Korea; 2 Department of Policy Development, National Development Institute of Korean Medicine, Seoul 04554, Republic of Korea; 3 Department of Public Health, College of Medicine, Korea University, Seoul 02841, Republic of Korea; 4 National Agency for Development of Innovative Technologies in Korean Medicine, National Development Institute of Korean Medicine, Seoul 07525, Republic of Korea

**Keywords:** symptoms of depression, sleep duration, Korea National Health and Nutrition Examination Surveys (KNHANES), national survey

## Abstract

This study aimed to determine the association between symptoms of depression and sleep duration in a representative sample of the Korean population. Using national cross-sectional data from the seventh Korea National Health and Nutrition Examination Surveys (KNHANES-VII), 5461 adults aged 18–49 years were analyzed using logistic regression models. The proportions of participants with total daily sleep durations (24 h) of <6 h, 6–8 h, and ≥9 h were 26.2%, 60.6%, and 13.3%, respectively. The proportions of individuals with symptoms of depression in the <6 h, 6–8 h, and ≥9 h sleep duration groups were 37.4%, 46.3%, and 16.3%, respectively. The odds ratios (ORs) were significantly higher in the <6 h and ≥9 h sleep groups than in the 6–8 h sleep group. There was a significant association between short (<6 h/day) and long (≥9 h/day) sleep duration and symptoms of depression among the general Korean population. In particular, our findings suggest that short sleep (<6 h/day) is more associated with symptoms of depression than long sleep (≥9 h/day).

## 1. Introduction

Sleep helps the human body eliminate physical and mental fatigue and maintain homeostasis. Additionally, it moderates the uncomfortable emotions experienced during the preceding day through dreams and information processing [1]. Therefore, sufficient sleep duration is a means for restoring the body’s energy and reducing stress and is, thus, a significant factor for achieving mental well-being.

Previous studies that analyzed the relationship between sleep duration and health revealed that insufficient sleep increases the risks of hypertension [2], obesity [3], and metabolic disorders [4]. Moreover, some studies have reported that the morbidity and fatality rates of cardiovascular diseases exhibits a U-shaped relationship with sleep duration [5]. Furthermore, chronic sleep insufficiency is accompanied by excessive drowsiness and low efficiency during the day, while compromising the functioning of the frontal lobe, causing cognitive disorders and reduced performance. Chronic sleep insufficiency also correlates with depression, which reportedly increases the prevalence of risky behaviors, accidents, and injuries [6].

According to a report published by the Organisation for Economic Co-operation and Development (OECD) in 2016, the average sleep time of adults or children living in South Korea was 7 h 51 min, 30 min shorter than the OECD average of 8 h 22 min. As a result of a survey of 18 OECD countries, Korea had the shortest amount of sleep (OECD, 2015). According to a global sleep survey conducted for World Sleep Day in 2021 with 13,000 participants from 13 countries, the sleep satisfaction rate of South Koreans was lower than the world average, with their average amount of sleep being 6.7 h during weekdays and 7.4 h during weekends, which was also short [7].

Depressive disorders have a high morbidity worldwide, as well as in South Korea, and the social cost of mood disorders is increasing; mood disorders generally manifest as negative impressions of oneself in the form of worrying, powerlessness, sense of loss, and feelings of worthless [8]. According to data published by South Korea’s Disease Control and Prevention Agency in 2017, depressive disorder was the most common psychiatric morbidity in 5.6% of the total population, and women had a higher burden of diseases than men, which is a measure of the impact of living with illness and injury and dying prematurely [9,10].

According to studies on depression and sleep, insomnia symptoms are associated with depression, while insomnia itself is a risk factor for depression [11]. Thus, when a person gets insufficient sleep, it negatively affects that person’s behavior, cognition, mood, and other aspects of daily life. An irregular sleep pattern can negatively affect physical and mental health while increasing the risk of accidents, reducing productivity, and causing social/relational issues [12].

In this study, we used data from the seventh Korea National Health and Nutrition Examination Survey (KNHANES-VII), which is representative of the total population of South Korea to quantitatively analyze the relationship between the sleep duration of the adult population and the occurrence of depression. The specific purposes of the study were as follows: (1) to examine the demographic data and health status of the sample and presence of depressive disorders in the sample in light of vocational factors; (2) to examine the relationship between sleep duration and occurrence of symptoms of depression among adult South Koreans.

## 2. Materials and Methods

### 2.1. Data Sources

Data were obtained from the KNHANES-VII (National Statistics Approval Number: 117002), which is a legal nationwide health and nutrition survey, in accordance with Article 16 of the National Health Promotion Act, conducted to determine the health status, morbidity rates for chronic diseases, and food and nutrient intake levels of the South Korean population. The national survey was initially meant to be conducted over three phases, starting with Phase 1 in 1988 to Phase 3 in 2009, when its frequency was revised to annual survey. Since Phase 4 (2007–2009), it has been conducted annually, as approved by the Institutional Review Board of the Korea Centers for Disease Control and Prevention (KCDC). These surveys have been conducted for the purpose of producing reliable and representative national statistics on the health of the general population, including the health status and intake of food and nutrients, and the statistical data are used for setting the goals of the General Plan for National Health Promotion, developing various health promotion programs, and informing health policies.

### 2.2. Sample Selection

**Figure 1:**
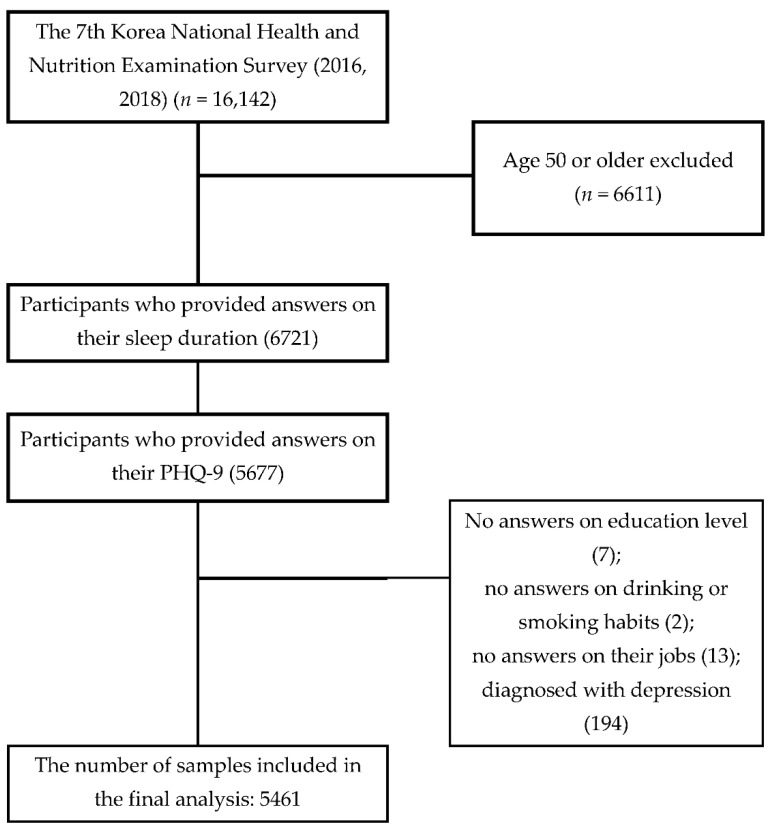
Flowchart of the sample selection. PHQ-9, Patient Health Questionnaire-9.

The data used in this study were collected from the KNHANES-VII (2016 and 2018). Of the 9531 participants, aged 18 to 49 years, 2810 who did not provide their sleep duration and 1044 who did not answer the question on symptoms of depression were excluded from the analysis, along with 7 who did not provide their level of education, 2 who did not answer the questions regarding drinking and smoking habits, 13 who did not specify their jobs, and 194 who were diagnosed with depression. This study focused on young and middle-aged people. In Korea, the government classifies those 18–49 as young and middle-aged, those between 50 and 64 as natural, and those over 65 as elderly. The selection process of the participants is shown in Figure 1.

### 2.3. Analysis Items

The items in the symptoms of depression analysis included demographics (sex, age, education level, and marital status), health behaviors (monthly drinking and smoking habits), and occupational factors (job, shifts, and sleep duration). KNHANES-Ⅶ used the Patient Health Questionnaire-9 (PHQ-9) as a screening measure to identify symptoms of depression. A PHQ-9 score of ≥10 was used to indicate the presence of symptoms of depression [13]. Participants diagnosed with depression were excluded from the study. In previous studies, a PHQ-9 score of ≥10 was used to determine the presence of symptoms of depression, and the reliability of this questionnaire has been proven [13]. The PHQ-9 items are based on the fourth edition of the Diagnostic and Statistical Manual of Mental Disorders and are designed to check for symptoms related to depression over the last 2 weeks. Each item is scored from 0 (not at all) to 3 (nearly every day). The PHQ-9, which consists of nine questions, is a reliable and valid depressive symptom scale. The total PHQ-9 score ranges from 0 to 27, with higher scores indicating greater severity of symptoms of depression [14,15]. In this study, the Cronbach’s alpha value for the PHQ-9 scale was 0.79. The relationship between symptoms of depression and sleep duration and demographic characteristics of 5461 questionnaires completed by 18–49-year-old South Koreans was analyzed.

Regular work shifts included day shifts (working during the day), nonregular shifts, and unemployed (housewives or students). Nonregular shifts included evening shifts (from 14:00 to 24:00), night shifts (21:00 to 08:00 the following day), regular alternating day–night shifts, 24-h shifts, divided shifts (working two or more shifts in a day), irregular shifts, and others.

The sleep time was recorded as the average daily sleep time (bed time: –hour –minute, wake up time: –hour –minute) calculated in minutes. Average sleep duration was measured and waking at night was not considered. Based on previous studies [16,17], we categorized sleep times as follows: short (<6 h of sleep per day), normal (6–8 h of sleep per day), and long (≥9 h of sleep per day).

### 2.4. Statistical Analysis

Basic analyses of the demographics, health behavior, and occupation factors and the impact of these factors on symptoms of depression were conducted using x-tests and logistic regression analyses. The data were analyzed using SAS 9.2 (SAS Institute Inc., Cary, NE, USA, 2011), and the significance level was set at 5%.

## 3. Results

### 3.1. Characteristics of Subjects

The demographic characteristics of the participants are presented in Table 1. The distribution of sleep durations between the sexes was as follows: men—<6 h, 27.1%; 6–8 h, 62.1%; ≥9 h, 10.8%; women—<6 h, 25.1%; 6–8 h, 58.9%; ≥9 h, 16.0%, showing a statistically significant difference (*p* < 0.0001). As the age increased, the sleep duration decreased (*p* < 0.0001). The distribution of sleep duration of <6 h increased with increasing age (<30 years, 24.3%; 30–39, 24.1%; ≥40 years, 29.3%); meanwhile, the distribution of sleep duration to ≥9 h decreased with increasing age (<30, 16.1%; 30–39, 15.0%; ≥40, 9.4%). Sleep durations of <6 h and 6–8 h were more frequently observed in the subgroup with an education level of “university graduate or higher” (26.2% and 61.8%, respectively). The duration of ≥9 h was more frequently observed in the subgroup with education level of “lower than university graduate” (14.8%) (*p* = 0.0218). As household income increased, the number of patients who slept ≥9 h decreased (low-income, 17.6%; mid-low, 15.1%; mid-high, 13.5%; high, 10.7%; *p* = 0.0104). Regarding the relationship between monthly drinking habits and sleep duration, those who had a monthly drinking habit were more likely to sleep 6–8 h a day (nondrinkers, 58.1%; drinkers, 61.8%) and <6 h (nondrinkers, 26.9%; drinkers, 25.8%). Nondrinkers (nondrinkers, 15.0%; drinkers, 12.4%) were more likely to sleep for at least 9 h (*p* = 0.0300). With regard to smoking habits, current smokers were more likely to sleep for <6 h (smokers, 28.9%; past smokers, 24.7%; nonsmokers, 25.4%), while fewer current smokers slept for 6–8 h (smokers, 58.6%; past smokers, 63.8%; nonsmokers, 60.4%) (*p* = 0.0498). Further, those who were employed were more likely to sleep for 6–8 h (employed, 63.0%; unemployed, 54.7%), while unemployed participants were more likely to sleep for ≥9 h (employed, 11.2%; unemployed, 18.1%) (*p* < 0.0001). Those working day shifts were more likely to sleep for 6–8 h (day shift, 64.5%; nonregular shift, 52.7%; unemployed, 53.3%). Those who worked nonregular shifts or were unemployed (housewives or students) were more likely to sleep for ≥9 h (day shift, 10.8%; nonregular shifters, 7.7%; unemployed, 18.1%) (*p* < 0.0001).

Those with symptoms of depression were more likely to sleep for <6 h (No, 25.5%; Yes, 41.6%) (*p* < 0.0001). Those with perceived stress were more likely to sleep for <6 h (no perceived stress, 24.0%; with perceived stress, 30.9%) and less likely to sleep for at least 9 h (no perceived stress, 14.5%; with perceived stress, 10.6%) (*p* < 0.0001) (Table 1).

### 3.2. Characteristics of the Subjects with and without Symptoms of Depression

The results of the cross-analysis between the demographic factors and symptoms of depression were as follows: The rate of symptoms of depression was higher in women (4.92%) than in men (2.88%) (*p* = 0.0013); the rates of symptoms of depression increased as the age decreased (<30, 4.92%; 30–39, 4.40%; ≥40, 2.74%) (*p* = 0.0130). The rates were statistically and significantly different among the smoker groups (smokers, 5.83%; past smokers, 3.66%; nonsmokers, 2.99%) (*p* = 0.0005); and the rates were higher in the unemployed group (employed, 2.83%; unemployed, 6.34%) (*p* < 0.0001). The tendency toward symptoms of depression was higher in the nonregular shift and unemployed groups (day shift, 3.19%; nonregular shift, 5.03%; unemployed, 5.16%) (*p* = 0.0130). The rate also increased if the sleep duration was <6 h or ≥9 h (<6 h, 6.13%; 6–8 h, 2.67%; ≥9 h, 4.75%) (*p* < 0.0001). Perceived stress also contributed to a higher tendency toward symptoms of depression (no perceived stress, 0.72%; perceived stress, 10.73%) (*p* < 0.0001) (Table 2).

### 3.3. The Factors That Affect Symptoms of Depression

The results of logistic regression analyses of the factors affecting symptoms of depression are shown in Table 3. In the Table 3, logistic regression analysis was performed. As a dependent variable, 0 was no depression, 1 was depressed. Model 1 was unadjusted; model 2 was gender and age corrected; model 3 was gender, age, education, household income, marital status, monthly drinking habits, smoking habits, job, shifts, perceived corrected.

In Model 1, the incidence of symptoms of depression was 2.381 times higher if the sleep duration was <6 h (*p* < 0.0001); the incidence was 1.821 times higher if the sleep duration was ≥9 h (*p* = 0.0006).

In Model 2, the incidence of symptoms of depression was 2.741 times higher if the sleep duration was <6 h (*p* < 0.0001) and 1.620 times higher if the sleep duration was ≥9 h (*p* = 0.0284). The incidence of symptoms of depression was higher in women than in men (odds ratio (OR) = 1.777, *p* = 0.0014). The incidence of symptoms of depression was lower among participants aged ≥ 40 years than among those aged under 30 (OR = 0.551, *p* = 0.0038).

In Model 3, the incidence of symptoms of depression was higher when the sleep duration was <6 h (OR = 1.979, *p* = 0.0010) and ≥9 h (OR = 1.849, *p* = 0.0099) than when it was 6–8 h. Women were more likely to have symptoms of depression (OR = 2.581, *p* < 0.0001). In addition, the high household income group was less likely to have symptoms of depression than the mid-high-income group (OR = 0.597, *p* = 0.0034). Nonmarried participants (OR = 1.765, *p* = 0.0286) and those who were separated/widowed/divorced (OR = 2.875, *p* = 0.0034) were more likely to have symptoms of depression than those who were married or in a common-law relationship. Current smokers (OR = 2.902, *p* < 0.0001) and past smokers (OR = 1.851, *p* = 0.0133) were more likely to experience symptoms of depression than nonsmokers. Unemployed participants (OR = 3.295, *p* < 0.0001) were more likely to experience symptoms of depression. Those working nonregular shifts (OR = 1.580, *p* < 0.05) and those with perceived stress (OR = 15.903, *p* < 0.0001) were more likely to have symptoms of depression.

## 4. Discussion

We used data from the 2016 and 2018 sections of the KNHANES-VII. We examined the sleep durations and incidence of symptoms of depression in 5461 adult participants (18 to 49) and the relationships between these variables.

Insufficient sleep (<6 h) and excessive sleep (≥9 h) both significantly associated with symptoms of depression. One study [18] analyzed the association between sleep duration and depression using the United States national survey data. Participants who had short (OR = 1.86) and long sleep (OR = 1.49) duration significantly associated with depression. Participants with short sleep duration (<5 and 5–6 h) had a higher risk of depression (OR = 1.69) and long sleep durations (>9 h) had no significant risks for depression compared to participants with normal sleep durations (7–8 h) [19]. Moreover, the meta-analysis [20] of seven prospective studies indicates that short and long sleep duration was significantly associated with increased risk of depression in 48,934 adults. This phenomenon can be explained by sleep debt, whereby insufficient sleep (<6 h) is accumulated like a debt, negatively impacting one’s health. Sleep debt reduces productivity and increases the risk of violent tendencies, leading to depression and suicidal issues [21]. People with mild symptoms of depression may oversleep to avoid this. This chronic oversleeping can lead to aggravation of depression. In other words, lack of sleep and too much sleep can both aggravate depression according to different mechanisms.

In this study of Dement et al. [21], the unemployed group had a significantly higher incidence of symptoms of depression than the employed group. In one study, a total of 21% of 24,553 workers aged between 25 and 65 years in 31 European countries suffered sleep disturbances in the last 12 months, and each unit increase in employment insecurity increased the odds of sleep disturbance by approximately 47% [22]. As such, job stress in the workplace and job insecurity may lead to a degradation of the quality of sleep and cause depression and anxiety. Therefore, caution is needed when interpreting the relationship between employment status and depression, and additional studies are needed to identify the direction of the causal relationship.

We found that women were 2.58 times more likely to experience symptoms of depression than men were, which is similar to the findings of existing studies [23,24]. In South Korea, the burden of housework and child rearing falls mainly on women [25,26]. Moreover, the amount of leisure time for women decreases as women spend more than 3 h and 10 min on housework (while men spend 48 min) [27]. Women are under heavy burden given their roles at work and at home. Due to conflicts arising from the need to play multiple roles and reduced leisure time, women have insufficient time to resolve their depression, which may be the reason why more women have symptoms of depression. In a study examining the relationship between job stress, quality of sleep, and symptoms among female and male workers [28], the incidence of depression was higher among women than among men, and the symptoms of depression were modified by job stress and quality of sleep; these results can be attributed to the increase in career-seeking and economic activities among women. Further studies on the relationship between gender difference and sleep duration are needed.

Regarding marital status, nonmarried (1.765 times) and separated/widowed/divorced (2.875 times) participants had a higher risk of developing symptoms of depression, while the incidence of depression was higher among younger participants, who are perceivably healthy, and current smokers, in line with the results of Lee et al. [29] Those with perceived stress had a 15.903-fold higher chance of developing symptoms of depression than those without. Stress increases the levels of corticosteroids in the bloodstream, causing the downregulation of glucocorticoid receptors; eventually, the ability to control emotions is reduced, making the stressed person vulnerable to depression. Therefore, stress is considered one of the key causes of depression [30,31], as highlighted in our study. Suh et al. [32] reported that insomnia was related to fatigue and depression, and this was supported by our results. As household income increased, the incidence of symptoms of depression decreased, in line with the findings of previous studies [33,34,35,36]. Low income increases negative experiences, such as resource deficiencies, while reducing positive experiences, such as self-control and social support; ultimately, access to psychological and social resources is reduced, and this negatively impacts mental health [37]. The low-income group tended to show a higher incidence of depression, possibly due to the high level of stress caused by various inconvenient experiences in their living environments [38].

Participants working nonregular shifts showed a higher incidence of symptoms of depression. Previous studies have reported that nonregular shifts may cause physical and mental health issues [39,40,41,42,43]. In one study, rotating shift was found to be five times more likely to cause shift work sleep disorder than regular shift, and individuals with shift work sleep disorder reported an increase in the levels of depression and anxiety symptoms [44]. The findings of this study are, in part, in line with the findings of previous studies, but further studies are needed to determine the symptoms or diseases caused by different types of nonregular shifts. On our study, current smokers were found to be 2.9 times more likely to experience depressive disorders. In a systematic review of 148 longitudinal studies [45], the results varied considerably, with evidence that smoking was associated with subsequent depression and anxiety and vice versa. Therefore, while it is still difficult to conclude that smoking causes depressive disorders, it is still believed that the findings of this analysis have a degree of credibility because they are based on Korean national data.

This study had several limitations. First, this was a cross-sectional study; thus, the causality between symptoms of depression and sleep duration could not be determined. Second, the KNHANES was conducted using a self-report questionnaire. The answers to the questions in our questionnaire could have been understated or overstated. Future studies including interviews and national check-ups are needed. Finally, although our results revealed that sleep duration was significantly associated with symptoms of depression, it is difficult to determine the exact sleep duration that is effective in preventing and improving symptoms of depression.

Despite these limitations, this study is of considerable value because it used large-scale nationwide data of the general Korean population to examine the relationship between sleep duration and symptoms of depression, and our findings are in line with those of previous studies [18,20].

## 5. Conclusions

In conclusion, we identified an association between the presence of symptoms of depression and short (<6 h/day) and long (≥9 h/day) sleep durations among the general Korean population, aged 18–49 years. Our findings suggest that sleeping less (<6 h/day) was associated with a higher incidence of depression than sleeping more (≥9 h/day). Our study indicate that women, nonregular shift workers, smokers, and individuals with perceived stress are at a greater risk of developing symptoms of depression, and these points should be kept in mind when counseling or coaching patients with depression. Moreover, future studies can examine other categories of sleep durations and the relationship between the quality of sleep and symptoms of depression so as to determine the accurate amount of sleep required to prevent or manage symptoms of depression.

## Figures and Tables

**Table 1:** Demographic distribution.

Category		Frequency	%	Sleep Duration			*p*
				Less Than 6 Hours *n* (%)	6–8 Hours *n* (%)	9 Hours or Longer *n* (%)	
Gender	Male	2435	52.5	654 (27.1)	1518 (62.1)	263 (10.8)	<0.0001
	Female	3026	47.5	751 (25.1)	1795 (58.9)	480 (16.0)	
Age	Under 30	1361	31.1	346 (24.3)	798 (59.5)	217 (16.1)	<0.0001
	30~39	1840	31.0	414 (24.1)	1126 (60.8)	300 (15.0)	
	40 and up	2260	38.0	645 (29.3)	1389 (61.2)	226 (9.4)	
Education	Less than university	2321	44.7	620 (26.1)	1345 (59.1)	356 (14.8)	=0.0218
	University graduate or higher	3140	55.3	785 (26.2)	1968 (61.8)	387 (12.0)	
Household income	Low	389	8.4	99 (23.9)	214 (58.5)	76 (17.6)	=0.0104
	Mid-low	1253	23.2	340 (27.2)	723 (57.7)	190 (15.1)	
	Mid-high	1872	34.0	465 (25.7)	1138 (60.8)	269 (13.5)	
	High	1947	34.5	501 (26.5)	1238 (62.8)	208 (10.7)	
Marital status	Not married	1796	39.3	471 (25.4)	1078 (61.0)	247 (13.5)	=0.8996
	Married (including common-law relationships)	3497	58.0	888 (26.6)	2136 (60.4)	473 (13.1)	
	Separated, windowed, or divorced	168	2.7	46 (28.3)	99 (58.4)	23 (13.3)	
Monthly drinking habits	Not drinking	1865	32.5	512 (26.9)	1075 (58.1)	278 (15.0)	=0.0300
	Drinking	3596	67.5	893 (25.8)	2238 (61.8)	465 (12.4)	
Smoking habits	Smoking	1262	25.9	348 (28.9)	748 (58.6)	166 (12.5)	=0.0498
	Smoked in the past	935	18.1	232 (24.7)	596 (63.8)	107 (11.5)	
	No smoking	3264	56.0	825 (25.4)	1969 (60.4)	470 (14.2)	
Job	Employed	3864	70.9	973 (25.8)	2445 (63.0)	446 (11.2)	<0.0001
	Unemployed	1597	29.1	432 (27.2)	868 (54.7)	297 (18.1)	
Shifts	Day shift	3597	65.7	852 (24.6)	2337 (64.5)	408 (10.8)	<0.0001
	Nonregular shifts	823	16.5	264 (29.7)	423 (52.7)	136 (17.7)	
	Unemployed (housewives, students)	1041	17.8	289 (28.5)	553 (53.3)	199 (18.1)	
Symptoms of depression	No	5247	96.2	1325 (25.5)	3214 (61.3)	708 (13.1)	<0.0001
	Yes (PHQ ≥ 10)	214	3.8	80 (41.6)	99 (42.0)	35 (16.4)	
Perceived	No	3747	68.8	889 (24.0)	2309 (61.5)	549 (14.5)	<0.0001
	Yes	1714	31.2	516 (30.9)	1004 (58.5)	194 (10.6)	

*p*: *p*-value, PHQ: Patient Health Questionnaire-9.

**Table 2:** A cross-analysis between the demographics and symptoms of depression.

Category		Without Symptoms of Depression(PHQ < 10) *n* (%)	With Symptoms of Depression (PHQ ≥ 10) *n* (%)	*p*
Gender	Male	2364 (97.12)	71 (2.88)	=0.0013
	Female	2883 (95.08)	143 (4.92)	
Age	Under 30	1294 (95.34)	67 (4.66)	=0.0130
	30–39	1757 (95.60)	83 (4.40)	
	40 and up	2196 (97.26)	64 (2.74)	
Education	Less than university	2213 (95.82)	108 (4.18)	=0.3083
	University graduate or higher	3034 (96.42)	106 (3.58)	
Household income	Low	362 (93.02)	27 (6.98)	=0.0003
	Mid-low	1186 (95.57)	67 (4.43)	
	Mid-high	1791 (95.79)	81 (4.21)	
	High	1908 (97.66)	39 (2.34)	
Marital status	Not married	1704 (95.15)	92 (4.85)	<0.0001
	Common-law relationship	3389 (97.06)	108 (2.94)	
	Separated, windowed, or divorced	154 (91.28)	14 (8.72)	
Monthly drinking habits	Not drinking	1798 (96.57)	67 (3.43)	=0.3177
	Drinking	3449 (95.95)	147 (4.05)	
Smoking habits	Smoking	1184 (94.16)	78 (5.84)	=0.0005
	Smoked in the past	896 (96.34)	39 (3.66)	
	No smoking	3167 (97.01)	97 (2.99)	
Job	Employed	3746 (97.17)	118 (2.83)	<0.0001
	Unemployed	1501 (93.66)	96 (6.34)	
Shifts	Day shift	3483 (96.81)	114 (3.19)	=0.0130
	Nonregular shifts	770 (94.96)	53 (5.04)	
	Unemployed (housewives, students)	994 (94.83)	47 (5.17)	
Sleep duration	Short (<6 h/day)	1325 (93.87)	80 (6.13)	<0.0001
	Normal (6–8 h/day)	3214 (97.33)	99 (2.67)	
	Long (≥9 h/day)	708 (95.25)	35 (4.75)	
Perceived	No	3721 (99.28)	26 (0.72)	<0.0001
	Yes	1526 (89.27)	188 (10.73)	

*p*: *p*-value, PHQ: Patient Health Questionnaire-9.

**Table 3:** The findings of the logistic regression analysis of the factors that affected symptoms of depression.

Category	Ref	Variable	Model 1				Model 2				Model 3			
			OR	95% Confidence Interval		*p*	OR	95% Confidence Interval		*p*	OR	95% Confidence Interval		*p*
				Lower Limit	Upper Limit			Lower Limit	Upper Limit			Lower Limit	Upper Limit	
Sleep duration	Normal (6–8 h/day)	Short (<6 h/day)	2.381	1.649	3.437	<0.0001	2.471	1.709	3.572	<0.0001	1.979	1.322	2.964	=0.0010
		Long (≥9 h/day)	1.821	1.189	2.789	=0.006	1.620	1.053	2.493	=0.0284	1.849	1.16	2.947	=0.0099
Gender	Male	Female					1.777	1.252	2.52	=0.0014	2.581	1.703	3.912	<0.0001
Age	Under 30	30~39					0.946	0.629	1.420	=0.7866	1.534	0.863	2.727	=0.1442
		40 and up					0.551	0.369	0.823	=0.0038	1.193	0.659	2.160	=0.5584
Education	University graduate or higher	Less than university									0.795	0.548	1.154	=0.2272
Household income	Mid-high	Low									1.347	0.730	2.487	=0.3395
		Mid-low									0.881	0.582	1.334	=0.5487
		High									0.597	0.373	0.957	=0.0322
Marital status	Common-law relationship	Not married									1.765	1.062	2.934	=0.0286
		Separated, windowed, or divorced									2.875	1.421	5.819	=0.0034
Monthly drinking habits	Not drinking	Drinking									0.815	0.562	1.182	=0.2792
Smoking habits	No smoking	Smoking									2.902	1.882	4.475	<0.0001
		Smoked in the past									1.851	1.138	3.011	=0.0133
Job	Employed	Unemployed									3.295	2.032	5.342	<0.0001
Shifts	Day shift	Nonregular shifts									1.580	1.035	2.411	=0.0341
		Unemployed (housewives, students)									0.696	0.391	1.240	=0.2183
Perceived	No	No									15.903	10.083	25.082	<0.0001

Findings in the logistic regression analysis; dependent variable: symptoms of depression (normal (0), with symptoms of depression (1)). Model 1: Only Sleep time; Model 2: Sleep time, gender, age; Model 3: Sleep time, gender, age, education, household income, marital status, monthly drinking, smoking habits, job shifts, perceived stress. OR: Odds Ratio, *p*: *p*-value, PHQ: Patient Health Questionnaire-9, ref: Reference.

## Data Availability

The Korea National Health and Nutrition Examination Survey (KNHANES) data used in this study can be obtained at https://knhanes.kdca.go.kr/knhanes/sub03/sub03_02_05.do after registration (accessed on 3 October 2021).
